# Prevention of infection in asplenic adult patients by general practitioners in France between 2013 and 2016: Care for the asplenic patient in general practice

**DOI:** 10.1186/s12875-020-01237-3

**Published:** 2020-08-12

**Authors:** Charlotte Quéffélec, Louis Billet, Pierre Duffau, Estibaliz Lazaro, Irène Machelart, Carine Greib, Jean-François Viallard, Jean-Luc Pellegrin, Etienne Rivière

**Affiliations:** 1 Internal Medicine and Infectious Diseases Unit, Haut-Leveque Hospital, University Hospital Centre of Bordeaux, F33600 Pessac, France; 2 Medical Information Department, Pellegrin Hospital, University Hospital Centre of Bordeaux, F33076 Bordeaux, France; 3 Internal Medicine and Clinical Immunology Unit, Saint-André Hospital, University Hospital Centre of Bordeaux, F33000 Bordeaux, France; 4 UMR CNRS 5164, ImmunoConcEpT & FHU ACRONIM, Bordeaux University, F33000 Bordeaux, France; 5 INSERM U1034, Bordeaux University, F33604 Pessac Cedex, France

**Keywords:** Splenectomy, General practice, Infections, Vaccination

## Abstract

**Background:**

Guidelines that detail preventive measures against *Streptococcus pneumoniae*, *Neisseria meningitidis*, *Haemophilus influenzae* type b, and influenza are published annually in France to decrease the risk of severe infections in immunocompromised patients. We aimed at describing adherence to these guidelines by GPs in the management of their asplenic patients in France between 2013 and 2016.

**Method:**

We conducted a multicenter retrospective study between January 2013 and December 2016 in three French hospitals: asplenic adults were identified and their GPs were questioned. A descriptive analysis was performed to identify the immunization coverage, type and length of antibiotic prophylaxis, number of infectious episodes, and education of patients.

**Results:**

103 patients were finally included in this study: only 57% were adequately vaccinated against *Streptococcus pneumoniae* or *Neisseria meningitidis*, 74% against *Haemophilus influenzae* type b, and 59% against influenza. Only 24% of patients received a combination of all four vaccinations. Two-thirds of patients received prophylactic antibiotics for at least 2 years. Overall, this study found that 50% of splenectomized patients experienced at least one pulmonary or otorhinolaryngological infection, or contracted influenza.

**Conclusions:**

These data match those reported in other countries, including Australia and the United Kingdom, meaning a still insufficient coverage of preventive measures in asplenic patients. Improved medical data sharing strategies between healthcare professionals, along with educational measures to keep patients and physicians up to date in the prevention of infections after splenectomy would improve health outcomes of asplenic patients.

## Background

In France, approximately 9000 patients underwent a splenectomy in 2016 due to trauma, lymphoid or myeloid neoplasm, autoimmune cytopenia, or in search of a diagnosis [1]. In addition to an increased thrombotic risk, asplenic patients also have a high risk of developing severe infections due to the roles of the spleen in blood filtration and in adaptive immunity. The most serious type of infection, caused by encapsulated bacteria, has a mortality rate of 50% within 48 h and is known as overwhelming post-splenectomy infection (OPSI) [2, 3]. Due to the presence of a specialized population of B lymphocytes in the splenic marginal zone that produce IgMs specific for the TI-2 protein of the polysaccharide capsule of OPSI causing bacteria, the spleen has the ability to eliminate these opsonization-resistant pathogens [3, 4].

Although the infectious risk in asplenic patients is high during their entire life, it is highest during the first 2 years following splenectomy and decreases over time. Therefore, national [5] and international guidelines [6–9] are regularly published to limit this infectious risk in asplenic patients by regularly up-dating the vaccination or antibiotic prophylaxis. For pneumococcal vaccination, the prime-boost strategy combines a 13-valent conjugate vaccine (pneumococcal conjugate vaccine-13, or “PCV13”) with a 23-valent-polysaccharide vaccine (pneumococcal polysaccharide vaccine-23, or “PPSV23”) 2 months after, then once every 5 years. In case of emergent splenectomy, PCV13 should be administered as soon as possible after surgery (ideally 15 days after, but sooner if the patient is at risk to be lost to follow-up), with PPSV23 2 months later. Vaccination against the main serogroups of *Neisseria meningitidis,* ACWY and B, comprises two injections either 6 months (for ACWY) or 1 month (for B) apart, with a recall against ACWY serogroups every 5 years. In addition, one injection of the *Haemophilus influenzae* type b (HIB) vaccine and one injection every year of the seasonal influenza vaccine are recommended. Furthermore, long-term prophylactic oral daily administration of an antibiotic, mainly phenoxymethylpenicillin, is required for at least 2 years after splenectomy to cover the period during which the infectious risk is highest [6–9]. General practitioners (GPs) have a central role in applying these preventive measures in collaboration with other physicians caring for the patient (oncologists, haematologists, internists, surgeons etc.). However, the institution of these preventive measures in asplenic patients appears insufficient, and there is also very heterogeneous post hoc management of infectious events [10–12]. Since the role of GPs is crucial in preventive and curative measures, we aimed to analyze the management of asplenic patients by GPs in accordance with published guidelines in France between the years of 2013 and 2016.

## Methods

A retrospective study was carried out in three French hospitals located in Bordeaux, Bayonne, and Pau, whose number of beds and chairs are respectively 3067, 1224 and 838. Adult patients (age ≥ 18) who underwent a splenectomy during the time period encompassing January 2013 to December 2016 were identified using the database in the three participating hospitals. Patients’ GPs were therefore questioned about the management of their asplenic patients, and this data collection was conducted between December 2017 and June 2018. GPs’ names were recorded in patients’ medical files. GPs were administered a questionnaire by phone to gather details about vaccinations against *Streptococcus pneumonia*, *Neisseria meningitidis*, HIB, and influenza virus, and prescription of prophylactic antibiotics, management of infectious events (mainly laboratory confirmed), patient’s possession of an emergency card, and information delivered to the patients. This questionnaire is available as Supplemental file 1. In our study, no GP took care of more than one asplenic patient, and all were aware of the splenectomy performed in their patient.

Exclusion criteria were established to exclude the following groups of asplenic patients: 1) non-adult patients, 2) patients who died of any cause between their splenectomy and the onset of data collection, 3) patients with partial splenectomy, 4) patients with functional asplenism, 5) patients without a GP, 6) patients whose GPs refused to participate or did not respond; 7) patients who had changed GPs, and 8) patients lost to follow-up. Medical records of included asplenic patients were utilized to gather information about prophylactic measures initiated before and after splenectomy by specialists other than the patients’ GPs, as well as any postoperative complications.

The questionnaire was registered at the National Commission on Informatics and Liberty (CNIL) in France (#MR-00313810*01, December 2017) and scrutinized for validation and confidentiality of collected data; our institutional review board also approved research. A descriptive analysis was then performed to identify the immunization coverage and the type and length of antibiotic prophylaxis. A univariate analysis assessed the effect of the cause underlying the splenectomy on the administered preventive measures. Chi-squared and Fisher’s exact test were used to statistically interrogate the collected data. All statistical analyses were performed with RStudio® software, with statistical significance defined as *p* < 0.05.

## Results

**Fig. 1:**
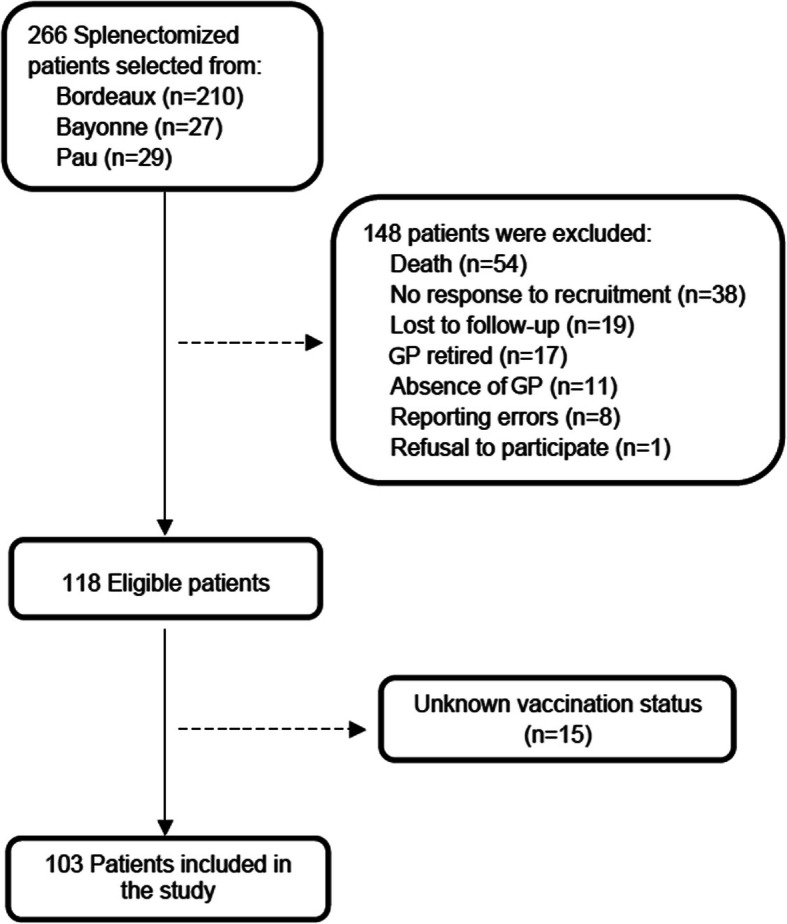
Flow Chart of the Study Population

We found that at the three chosen hospitals, 266 patients were splenectomized between January 2013 and December 2016. Among these patients, 163 were excluded based on the criteria stated in the Methods section and summarized in Fig. 1. For the remaining 103 patients, we gathered data on the general characteristics, cause of the splenectomy and duration of follow-up by GPs in the medical files (Table 1). Then, patients were divided into 4 groups based on the precipitating cause for their splenectomy: 1) trauma and iatrogenesis, 2) malignancy, 3) autoimmune cytopenia, and 4) other (Table S1). Trauma and idiopathic thrombocytopenic purpura were the most common causes of splenectomy (*n* = 44). We then called patients’ GPs to gather additional information about their care to their asplenic patient during a mean follow-up period of 3.5 years (Table 1).

**Table 1:** Patient Characteristics

	Number of patients
Gender	
Male	52
Female	51
Age, years	
Median (range)	57 (20–84)
Mean	55
Cause of splenectomy	
Trauma and iatrogenesis	31
Malignancy	28
Autoimmune cytopenia^a^	23
Other	21
Follow-up time, years	
< 2	25
2–3	23
3–4	34
> 4	21
Mean	3.5

^a^Autoimmune cytopenias were idiopathic thrombocytopenic purpura (ITP) and autoimmune haemolytic anaemia (AIHA)

First, we analyzed preventive measures. Importantly, only 24% adequately received all recommended vaccinations combined (Table 2). Overall, 87% received at least one injection of a pneumococcal vaccine, and 57% received the necessary pneumococcal vaccination booster. Since boosted vaccination against *Streptococcus pneumoniae* was introduced in the 2014 guidelines in France, we noted that 76% of splenectomized patients in 2016 received the boosted pneumococcal vaccination compared to 52% in 2013 (*p* = 0.0005). Univariate analysis revealed that patients with autoimmune cytopenia were significantly more vaccinated against *Streptococcus pneumoniae* compared to the other three groups (*p* = 0.038, Table S2). Vaccination rates against other germs are summarized in Table 2.

**Table 2:** Patient Vaccination Details

Strains	Vaccinated	Not vaccinated
Pneumococcal	***n*** **= 90 (87.38%)**	***n*** **= 13 (12.62%)**
**Adequate PCV13/PPSV23 vaccination schedule**	*n* = 59 (57.28%)	
**Inadequate PCV13/PPSV23 vaccination schedule**	*n* = 5 (4.85%)	
**PCV13 only**	*n* = 6 (5.83%)	
**PPSV23 only**	*n* = 20 (19.42%)	
Meningococcus (by serogroups)	***n*** **= 59 (57.28%)**	***n*** **= 44 (42.72%)**
**C**	*n* = 24 (23.30%)	
**AC**	*n* = 10 (9.70%)	
**ACWY**	*n* = 20 (19.42%)	
**B**	*n* = 1 (0.98%)	
**C + B**	*n* = 2 (1.94%)	
**ACWY + B**	*n* = 2 (1.94%)	
*Haemophilus influenzae* B	***n*** **= 77 (74.76%)**	***n*** **= 26 (25.24%)**
Annual Influenza	***n*** **= 61 (59.22%)**	***n*** **= 42 (40.78%)**
All vaccinations, ***ie adequate PCV13/PPSV23 + ACWY/B + HIB + influenza vaccination schedules***	***n*** **= 24 (23.30%)**	***n*** **= 79 (76.70%)**

**Fig. 2:**
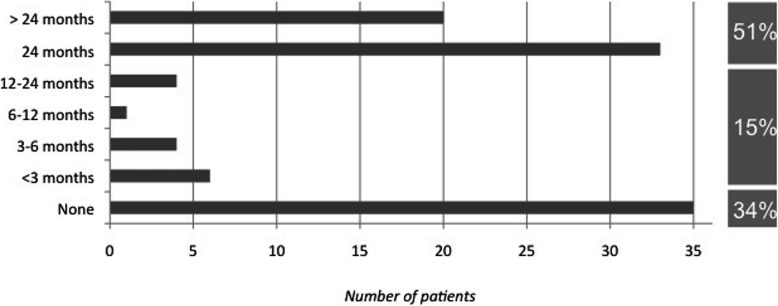
Duration of Antibiotic Prophylaxis

Antibiotic prophylaxis was administered in 68 of 103 patients (66%). However, prescription duration was heterogeneous as shown in Fig. 2. GPs reported that the absence of antibiotic prophylaxis and a short prescription duration of under 2 years in 45 patients were both linked to oversight or unfamiliarity with established guidelines. Long-term prescriptions of over 2 years were either given by specialized physicians for patients with haematological malignancies or solid neoplasms (*n* = 8), or due to the unawareness of physicians that established guidelines limited prescription duration to 2 years (*n* = 11). Three patients had an allergic reaction to the prescribed antibiotic, so drug administration was stopped without prescription of a new antibiotic. Oral penicillin V was used in 62 patients while 6 patients received another antibiotic (amoxicillin in 4 patients, and erythromycin in 2 patients).

Next, we detailed infectious episodes reported by GPs between 2013 and 2016. Of note, 47 patients (45%) developed at least one infection: 24 (23%) had one infectious episode, 12 (11%) two, 2 (2%) three, and 9 (8%) more than three; 56 patients had no infectious episode. The majority of these were pulmonary or ear-nose and throat infections, isolated fevers, or flu (Table S3). Notably, 4 patients who had not been previously vaccinated or receiving antibiotic prophylaxis had an overt OPSI in the month following splenectomy. Of these, 3 patients had reversible respiratory failure and one patient had urinary and intra-abdominal infections with bacteraemia and septic shock. Two other patients were tested positive for additional pathogens including *Escherichia coli, Pseudomonas aeruginosa, Enterobacter cloacae, and methicillin-resistant Staphylococcus aureus* while under postoperative care. In addition to these 6 patients, 7 others had to be hospitalized to manage an infection; however, a complete diagnostic assessment was only carried out in 28% of all infectious cases.

Then, we analyzed the main infections in the 31 asplenic patients who received vaccinations. The number of infectious episodes due to pathogens potentially covered by the vaccines (pulmonary, otorhinolaryngological, and influenza infections due to *Streptococcus pneumoniae, Haemophilus type B,* or influenza virus) are shown in Table 3. Of note, these patients were very heterogeneously vaccinated, and half were receiving antibiotic prophylaxis. In addition, these infectious episodes were present in different proportions of patients according to the cause of splenectomy with the following distribution: 9.7% with autoimmune cytopenias, 22.6% with trauma and iatrogenesis, 29% with malignancy, and 38.7% with other causes. Thus, the data in our study suggest that patients with autoimmune cytopenia had fewer episodes of pulmonary, ear nose throat, and influenza infections. On the other hand, patients with malignancies had more infectious episodes. Specifically, six out of 9 patients experiencing more than 3 infectious episodes had lymphoma. No patients had meningococcal infection.

**Table 3:** Infections Due to Bacteria with Potential Coverage by Vaccines or Antibiotic Prophylaxis

➔ Preventive ongoing measures ➔	In asplenic patients vaccinated with pneumococcal vaccines			In asplenic patients vaccinated with HIB vaccine	In asplenic patients vaccinated with annual influenza vaccine	In asplenic patients vaccinated with ***Neisseria meningitidis*** vaccines (all serogroups)	In asplenic patients receiving antibiotic prophylaxis ≥2 years
	with PCV13 only	with PPSV23 only	with PCV13+PPSV23				
**Number of infections** ***(lung*** ***and/or ENT*** ***and/or influenza)*** **(*N*** **= 31)**	2	9	16	22	21	14	18

*ENT* ear-nose-throat, *PCV13* pneumococcal conjugate vaccine-13, *PPSV23* pneumococcal polysaccharide vaccine-23, *HIB* Haemophilus influenza type B

Finally, we assessed how patients were informed about their increased risk for infection. Patients only received oral information about the global risks of the splenectomy, and in 74% of cases, this information was delivered by hospital physicians. As indicated by GPs, we found that 16 patients had an emergency alert card without knowing its precise origin or type.

## Discussion

Our results show that asplenic patients are not adequately protected against the common pathogens that are targeted by vaccination and antibiotic prophylaxis in clinical practice: only 24% of patients received all recommended vaccines and 66% received adequate antibiotic prophylaxis.

Of note, the rate of flu vaccination was probably underestimated by GPs since nurses are authorized to independently vaccinate against influenza in France since 2008.

We confirm in our study that the risk of infection remains high in asplenic patients, even in patients receiving prophylactic antibiotics, especially in case of haematological disease, neoplasm, or older age [13, 14]. We identified 83 infectious events, mainly pulmonary or ENT infections and influenza, reported by GPs in 47 patients. This is likely an underestimation since patients could have potentially consulted another healthcare professional, such as physicians in emergency units. Lastly, we identified 4 occurrences of OPSI in the month following splenectomy in patients who did not receive any vaccine or antibiotic prophylaxis.

Recent work in other regions of France and Australia also showed a low pneumococcal vaccination rate of 18.8 and 7%, respectively [10, 15]. Similar to our study, international studies have reported a pneumococcal vaccination rate between 60 and 88% [10, 12, 16–23]. Our study shows, however, that only 57% of patients received the adequate booster. An increased rate of boosted anti-pneumococcal vaccination was observed only in medical records from 2016, despite the published recommendation for a booster schedule in 2014 in France. This indicates a notable delay in implementation by physicians. Of note, patients with autoimmune cytopenias had the most number of vaccinations against *Streptococcus pneumoniae* compared to the other groups, likely because these patients were more frequently vaccinated prior to the administration of necessary immunosuppressive agents to combat their autoimmune disorder [24].

Regarding vaccination against *Neisseria meningitides*, the vaccination rate was higher in our study than in other previously published results [10, 23, 25], as was the rate of vaccination against HIB [10, 16, 18, 22, 23, 25]. Of note, the low vaccination rate against B serogroup can be partly explained by the evolution of guidelines in France, as the anti-MenB vaccine was recommended for asplenic patients in 2013 and reimbursed by the Sécurité Sociale in December 2014. However the vaccination rate against MenB still remains low. Finally, the proportion of asplenic patients receiving antibiotic prophylaxis is slightly lower than previously reported [10, 13, 26, 27].

Our study describes the medical management of asplenic patients by GPs, adding valuable insights to an otherwise scarce body of work. The total number of patients analysed exceeds other similar international studies. Nevertheless, our study has several limitations. Of note, the data from 166 patients could not be analyzed, mostly because their were deceased, lost to follow-up, or because their GP did not answer to our solicitations or had retired at the time of the study. We also identified four biases inherent to the chosen methodology of research: 1) a non-response bias due to GPs who did not want to participate, 2) selective survival bias linked to the presence of many deceased asplenic patients within the chosen timeframe, 3) recall bias, especially for collecting information about patients’ education through GPs, and 4) storage bias. In addition, the questionnaire was not designed to assess patients’ compliance with the prescribed schedule/dosage of antibiotic prophylaxis, timing of vaccination in patients receiving immunosuppressive therapies, effective therapeutic education of the patient, patients’ general knowledge regarding asplenic states, or availability of a curative antibiotic without a prescription. Finally, timing of this study has not indexed PPSV23 recall at 5 years.

### Implications for research and/or practice

#### How to increase the infectious prophylaxis in asplenic patients in general practice?

A dedicated healthcare network has been reported useful for the follow-up of asplenic patients in several countries [21, 28–32]. Moreover, creating nationwide registries of asplenic patients has correlated with improved implementation of established guidelines among physicians and allowed for the dissemination of useful information, medical advice, and vaccination reminder in a cost-effective manner [15, 26, 33]. We insist on the central role of hospital specialists taking care of the patient before and at the time of splenectomy for starting the preventive measures against infection, and beginning patient’s medical education about his future asplenic state. Furthermore, the ongoing implementation of a shared medical record system in France will certainly be useful to synchronize asplenic patients care in the future. Finally, GPs are crucial to coordinate care for patients with chronic blood diseases starting an infectious episode, in cooperation with hematologists. Helpful measures for physicians to improve care of the asplenic patient are summarized in Table 4.

**Table 4:** Measures to Improve Care of Asplenic Patients in Healthcare Practice (adapted from [3, 6–8, 34, 35])

**Vaccinate against commonly encountered encapsulated bacteria and influenza** *(guidelines may vary according to each country)*	***Streptococcus pneumoniae***
	PCV13 then PPSV23 2 to 12 months later (boosted strategy) − consider another boosted strategy if PPSV23 has been injected more than one year after PCV13 − consider adding PCV13 if PPSV23 was given first, and do a new boosted vaccination 5 years after the PPSV23 injection − respect a 5-year minimum interval between two doses of PPSV23
	***Neisseria meningitidis***
	Consider two doses of tetravalent ACWY vaccine in a 6-month interval, and a recall every 5 years Consider two doses of anti-MenB vaccine in a 1-month interval
	***Haemophilus influenzae*** **type b**
	Consider one dose of the vaccine in adulthood
	**Influenzae**
	Consider an annual dose of vaccine in November
**Antibiotic prophylaxis**	**Oral penicillin V, or erythromycin in case of allergy, for at least two years**
	- consider lifelong prophylaxis in patients at high risk: age < 16 or > 50, survival to a first OPSI episode, patients with haematological diseases, HIV, or ongoing immunosuppressive therapies, or inadequate response to pneumococcal vaccination - reconsider this attitude regarding the evolution of local bacterial ecology and patient’s medical history or concomitant medications over time (drug interactions or contra-indications)
**Therapeutic education**	**Educate patients with recurrent information about:**
	- the function of the spleen - the infectious risk: encapsulated bacteria, the role of influenza in such infections, alert signs of infection, how to act at signs of infection - vaccinations to be done over time - antibiotic prophylaxis - animal and tick bites - communication of the asplenic state to other healthcare professionals - medical ID (splenectomy card or personalized medical ID) - travel advice

It is important to note that, despite good vaccination coverage, asplenic patients remain at risk for infection, notably those not covered by vaccines such as enteritis or cold. However, adequate vaccination against encapsulated bacteria will limit the risk of severe infectious complications.

#### How to improve patient education before and after splenectomy?

Despite the advent of vaccination and antibiotic prophylaxis, ongoing guidelines are not adequately implemented in clinical practice. Our study shows that patients are not well enough informed regarding the infectious risk associated with their asplenic state [16, 36–38] while it has been shown that patient’s education can reduce the risk of OPSI [37]. Our data suggest that improved communication between healthcare professionals can decrease the incidence of infection in these patients. In fact, 13% of patients were excluded from our study because their GPs were unaware or uncertain about the patients’ anti-infectious prophylactic measures. In addition, the majority of patients only received information orally from their physicians, but no written documents. In healthcare education, audiovisual support and printed documents have been shown to be more effective in strengthening medical education and the understanding of patients compared to oral information [16, 39, 40]. To that end, previous studies analyzed the tools and quality of written information available on the internet for asplenic patients to assess the quality of accessible information [41, 42]. These studies found that countries such as Australia [43] and United Kingdom [44] provide dedicated websites to asplenic patients in addition to information leaflets and patient emergency cards. It is clear that standardization of educational material regarding asplenic states is needed in other countries.

## Conclusions

Our study shows that asplenic patients are insufficiently protected against encapsulated bacteria. The role of GPs is central in long-term monitoring and management of infectious events in this population of patients, in collaboration with all healthcare professionals. Guideline implementation must be improved through adequate transmission of information between healthcare professionals, and iterative and complete education of both physicians and asplenic patients.

## Supplementary information

**Additional file 1.** 7-question questionnaire used by main investigator (CQ) to question GPs about their management of their asplenic patient.

**Additional file 2: Supplemental Table 1.** Detailed Indications for Splenectomy in the Study Population.

**Additional file 3: Supplemental Table 2.** Univariate Analysis of the Vaccinations Received According to the Cause of Splenectomy.

**Additional file 4: Supplemental Table 3.** Notification of Infections by GPs.

## Data Availability

The datasets used and/or analysed during the current study available from the corresponding author on reasonable request.

## Abbreviations

GP: General practitioner
OPSI: Overwhelming post-splenectomy infection
PCV-13: Pneumococcal conjugate vaccine-13
PPSV23: Pneumococcal polysaccharide vaccine-23
HIB: *Haemophilus influenzae* type B
CNIL: National Commission on Informatics and Liberty
ENT: Ear-nose-throat
