# Supplementary material for: Prevention of infection in asplenic adult patients by general practitioners in France between 2013 and 2016: Care for the asplenic patient in general practice
Source: BMC Fam Pract. 2020 Aug 12;21:163. doi: 10.1186/s12875-020-01237-3 (PMC7425533; doi:10.1186/s12875-020-01237-3)
Supplement: Supplementary file 1 — Additional file 1. 7-question questionnaire used by main investigator (CQ) to question GPs about their management of their asplenic patient. [file 12875_2020_1237_MOESM1_ESM.docx]

**Prevention of Infection in Asplenic Patients by General Practitioners in France Between 2013 and 2016.**

**Appendix 1**

**7-question questionnaire used by main investigator (CQ) to question GPs about their management of their asplenic patient.**

| **QUESTIONS** | **CUES** |
| --- | --- |
| 1/ Have your patient received pneumococcal vaccine? | If YES:   - Which(s) pneumococcal vaccine(s)?   (“boosted” scheme?)   - Vaccination date?   If NO:   - Why? (Oversight? Patient refusal? Allergy? GP refusal? Out of stock ?) |
| 2/ Have your patient received meningococcal vaccine? |  |
| 3/ Have your patient received *heamophilus influenzae* type B vaccine? |  |
| 4/ Have your patient received anti-influenzae vaccine? |  |
| 5/ Have your patient received antibiotic prophylaxis against encapsulated bacteria infections? | If YES:   - Which(s) molecule(s)? - Dose? - Duration? (months)   If NO:   - Why? - Other option? |
| 6/ Did your patient experience one or more infectious episode after splenectomy? | If YES:   - How many and what were they? - What was the diagnostic work-up? - What treatment was administered?   If NO:   - Did your patient experience another complication ? |
| 7/ Does your patient have a splenectomy card? | YES  If NO: why? |
