# Supplementary material for: Prevention of infection in asplenic adult patients by general practitioners in France between 2013 and 2016: Care for the asplenic patient in general practice
Source: BMC Fam Pract. 2020 Aug 12;21:163. doi: 10.1186/s12875-020-01237-3 (PMC7425533; doi:10.1186/s12875-020-01237-3)
Supplement: Supplementary file 2 — Additional file 2: Supplemental Table 1. Detailed Indications for Splenectomy in the Study Population. [file 12875_2020_1237_MOESM2_ESM.docx]

**Supplemental Table 1. Detailed Indications for Splenectomy in the Study Population**

| Trauma | n=25 |
| --- | --- |
| Iatrogenicity | Surgical complication*, n=4  Thoracocentesis, n=2 |
| Non-malignant haematological diseases | Idiopathic thrombocytopenic purpura, n=19  Autoimmune haemolytic anaemia, n=4  Hereditary spherocytosis, n=2  Thrombocytopenia by hypersplenism, n=1  Constitutional thrombocytopenia, n=1 |
| Hematological malignancies | Non-Hodgkin lymphoma**, n=15  Chronic lymphocytic leukemia, n=1  Myelofibrosis, n=1  Granular lymphocyte leukemia, n=1 |
| Oncology | Ovarian cancer, n=3  Colorectal cancer, n=2  Gastric adenocarcinoma, n=1  Oesophageal cancer, n=1  Urothelial carcinoma, n=1  Pancreatic carcinoma, n=1  Malignant tumour of the peritoneum, n=1 |
| Other | Splenic cysts, n=5  Rupture of an aortic aneurysm, n=4  Splenic abscess, n=2  Mononucleosis, n=1  Spontaneous splenic rupture, n=1  Sickle cell disease, n=1  Niemann-Pick C disease, n=1  Sarcoidosis, n=1  Mesenteric ischemia n=1 |

** Initial surgery leading to a perioperative complication: Gastropleural fistula, n=1; post-surgery hematoma, n=1; peritonitis after sigmoid perforation, n=1; aortic stenosis, n=1*

***Non-Hodgkin lymphomas: Marginal zone, n=11; diffuse large B-cell, n=1; lymphocytic lymphoma, n=1; Burkitt n=1; follicular, n=1*
