# Supplementary material for: Prevention of infection in asplenic adult patients by general practitioners in France between 2013 and 2016: Care for the asplenic patient in general practice
Source: BMC Fam Pract. 2020 Aug 12;21:163. doi: 10.1186/s12875-020-01237-3 (PMC7425533; doi:10.1186/s12875-020-01237-3)
Supplement: Supplementary file 3 — Additional file 3: Supplemental Table 2. Univariate Analysis of the Vaccinations Received According to the Cause of Splenectomy. [file 12875_2020_1237_MOESM3_ESM.docx]

**Supplemental Table 2. Univariate Analysis of the Vaccinations Received According to the Cause of Splenectomy.**

| Cause of Splenectomy | Vaccination | | | | |  |
| --- | --- | --- | --- | --- | --- | --- |
|  | **Boosted SP** | **HIB** | **NM** | **Influenza** | **All vaccination**s |  |
|  | **N (%)**  **[p-value]** | | | | | |
| Trauma/  Iatrogenesis | 14 (13.59%)  [0.1572] | 20 (19.42%)  [0.1859] | 13 (12.62%)  [0.06449] | 18 (17.47%)  [1] | 4 (3.88%)  [0.1664] |  |
| Autoimmune cytopenia | 18 (17.48%)  [**0.03857**] | 20 (19.42%)  [0.2092] | 16 (15.53%)  [0.2661] | 10 (9.71%)  [0.1329] | 5 (4.85%)  [1] |  |
| Malignancies | 17 (16.50%)  [0.8364] | 22 (21.36%)  [0.7722] | 17 (16.51%)  [0.8364] | 20 (19.42%)  [0.1886] | 9 (8.74%)  [0.3007] |  |
| Others | 10 (9.71%)  [0.4496] | 15 (14.56%)  [0.9108] | 13 (12.62%)  [0.8159] | 13 (12.62%)  [0.9749] | 6 (5.83%)  [0.7256] |  |

*SP, Streptococcus pneumoniae; NM, Neisseria meningitidis; HIB, Haemophilus influenzae type b*
