# Supplementary material for: Prevention of infection in asplenic adult patients by general practitioners in France between 2013 and 2016: Care for the asplenic patient in general practice
Source: BMC Fam Pract. 2020 Aug 12;21:163. doi: 10.1186/s12875-020-01237-3 (PMC7425533; doi:10.1186/s12875-020-01237-3)
Supplement: Supplementary file 4 — Additional file 4: Supplemental Table 3. Notification of Infections by GPs. [file 12875_2020_1237_MOESM4_ESM.docx]

**Supplemental Table 3. Notification of Infections by GPs.**

| Infection Type | Infectious events  N | Diagnostic evaluation  N | Antibiotic treatment  N | Hospitalization  N |
| --- | --- | --- | --- | --- |
| Lung | 25 | 10 | 21 | 1 |
| ENT | 25 | 4 | 23 | 0 |
| Urinary | 11 | 7 | 11 | 2 |
| Fever syndrome/Flu | 7 | 3 | 3 | 2 |
| Skin | 9 | 1 | 6 | 0 |
| Gastrointestinal | 2 | 0 | 2 | 0 |
| Intravascular device | 2 | 1 | 2 | 1 |
| Dental | 1 | 1 | 1 | 0 |
| Bone infection | 1 | 1 | 1 | 1 |
| *Total* | ***83*** | ***28*** | ***70*** | ***7*** |
